# On the Development of Parafoveal Preprocessing: Evidence from the Incremental Boundary Paradigm

**DOI:** 10.3389/fpsyg.2016.00514

**Published:** 2016-04-14

**Authors:** Christina Marx, Florian Hutzler, Sarah Schuster, Stefan Hawelka

**Affiliations:** Centre for Cognitive Neuroscience, University of SalzburgSalzburg, Austria

**Keywords:** reading fluency, reading acquisition, eye movement control during reading, incremental boundary paradigm, visual word recognition

## Abstract

Parafoveal preprocessing of upcoming words and the resultant preview benefit are key aspects of fluent reading. Evidence regarding the development of parafoveal preprocessing during reading acquisition, however, is scarce. The present developmental (cross-sectional) eye tracking study estimated the magnitude of parafoveal preprocessing of beginning readers with a novel variant of the classical boundary paradigm. Additionally, we assessed the association of parafoveal preprocessing with several reading-related psychometric measures. The participants were children learning to read the regular German orthography with about 1, 3, and 5 years of formal reading instruction (Grade 2, 4, and 6, respectively). We found evidence of parafoveal preprocessing in each Grade. However, an effective use of parafoveal information was related to the individual reading fluency of the participants (i.e., the reading rate expressed as words-per-minute) which substantially overlapped between the Grades. The size of the preview benefit was furthermore associated with the children’s performance in rapid naming tasks and with their performance in a pseudoword reading task. The latter task assessed the children’s efficiency in phonological decoding and our findings show that the best decoders exhibited the largest preview benefit.

## Introduction

While our eyes move across continuous texts in a sequence of fixations, we extract information not only from the word which we are currently fixating, but also from the not-yet fixated, upcoming word (Rayner, 1998). This parafoveal preview gives us first orthographic and phonological (and potentially lexical) information about the upcoming word (Schotter et al., 2012). Parafoveal preprocessing therefore accelerates foveal word recognition and hence contributes to fluent reading. Evidence regarding the developmental trajectory of parafoveal preprocessing, however, is limited.

Two gaze-contingent techniques are commonly used for investigating parafoveal preprocessing: (i) the *moving window paradigm* (McConkie and Rayner, 1975) and (ii) the *invisible boundary paradigm* (Rayner, 1975). Within the moving window paradigm, a text outside a predefined “window” to the left and right of the current fixation is masked, for example, by *X*s. The text within the window is presented unmutilated. By means of this paradigm, a reader’s perceptual span can be estimated, that is, the minimal window size by which the reader is not affected by the parafoveal masks. Research using this paradigm demonstrated that the perceptual span for adult readers ranges from 3 to 4 letters left and 14 to 15 letters right of fixation (e.g., McConkie and Rayner, 1975). By contrast, the perceptual span of beginning readers undergoes development, that is, it increases with reading experience. To illustrate, 2nd and 4th Grade children have a smaller span compared to adults, that is, about 3–4 letters to the left and about 11 letters to the right of fixation. Children from Grade 6, however, already show an adult-like span size (Rayner, 1986; Häikiö et al., 2009; Sperlich et al., 2015). In sum, evidence from the moving window paradigm suggests that children utilize information beyond the currently fixated word.

The most commonly used technique to study effects of parafoveal preprocessing of the upcoming word is the invisible boundary paradigm (Rayner, 1975). Within this paradigm an invisible boundary is placed before a theoretically relevant target word. As long as the reader fixates to the left of the boundary, a valid or an experimentally manipulated preview is presented (e.g., a X-mask, that is, a string of *X*’s preserving the length of the target word or a same-shape/different-letter mask, that is, a sequence of different letters preserving the target word’s length and shape). Contingent on crossing the boundary, the manipulated parafoveal preview is replaced with the target word. In order to estimate the preview benefit, fixation durations for valid previews are compared to those of manipulated (e.g., X-masked) previews. Research utilizing this paradigm showed – for adult, proficient readers – that the magnitude of the preview benefit is around 30–50 ms (Rayner, 2009).

Recent findings, however, indicated that the classical variant of the boundary paradigm does not provide an accurate estimate of the preview benefit (Hutzler et al., 2013; Kliegl et al., 2013; Marx et al., 2015). To be specific, when parafoveal masks are used as a baseline, they inflict processing costs and hence inflate the estimated preview benefit. A recent study from our lab revealed such an erroneous overestimation of the preview benefit in beginning readers (Marx et al., 2015). In the light of these recent findings, we adapted the classical approach and introduced the *incremental boundary technique* for investigating the development of parafoveal preprocessing in children (Marx et al., 2015). In short, instead of using parafoveal masks, we manipulated the salience of the parafoveal previews by gradually reducing its visual integrity (i.e., displacing a certain amount of pixels of the preview). In so doing, we can assess whether increasing salience leads to shorter processing times, that is, to a preview benefit (see Jacobs et al., 1995 for the logic of this within-condition baseline).

To date, three studies, which used the classical variant of the invisible boundary paradigm, provided evidence on parafoveal preprocessing in children. One study examined whether children from Grade 2, 4, and 6 extract information from a second constituent of a compound word (e.g., *ball* in *basketball*; the boundary was between *basket* and *ball*; Häikiö et al., 2010). This condition was compared to a condition which presented (space-separated) adjective–noun pairs (e.g., *little ball*). The authors reported that even 2nd Graders profited (in terms of shorter subsequent fixations) from “parafoveal” information when it was connected to the fixated word (i.e., the compound condition) compared to the adjective–noun condition. Another study examined whether 8 to 9-year-old children benefit from parafoveal phonological information (i.e., by presenting pseudohomophone previews) and orthographic information (i.e., by presenting transposed-letter previews; Tiffin-Richards and Schroeder, 2015). They found that children – in contrast to adults – showed a pseudohomophone preview benefit, that is, they profited from the availability of phonological information in the parafovea. The third study investigated – in 4th Graders and adults – the influence of available orthographic information in parafoveal vision by transposing the letters of the initial trigrams of the previews (Pagán et al., 2015). Interestingly, the authors found similar effects for both groups, that is, children and adults alike were able to preprocess orthographic information. In sum, evidence suggests that 2nd Graders use parafoveal information from the second noun in a compound word pair and also benefit from phonological information presented parafoveally (Häikiö et al., 2010; Tiffin-Richards and Schroeder, 2015). Regarding the orthographic aspect of parafoveal preprocessing, however, it is still unclear whether the transposed letter manipulation induced preview costs on its own and hence resulted in an overestimation of the preview benefit (as demonstrated in Marx et al., 2015, for same-shape/different-letter masks).

### The Association of Reading Fluency, Phonological Decoding, and Rapid Naming with Parafoveal Preprocessing during Reading

In addition to the development of parafoveal preprocessing, we were interested how the capability of using parafoveal information for subsequent foveal word recognition relates to reading fluency and the children’s performance in reading-related tasks. We therefore assessed the relationship between the children’s reading rate in the present sentence reading task and the estimated gain of parafoveal preprocessing. Additionally, we assessed the relationship between parafoveal preprocessing and the performance of reading lists of (unrelated) words and pseudowords. Reading pseudowords taps into the children’s efficiency of phonological decoding. The German orthography is very regular, that is, the grapheme–phoneme correspondence is highly consistent (in contrast to the irregular English orthography). Evidence suggests that the gain in reading fluency of children learning to read a regular orthography is primarily due to a more efficient phonological (i.e., sublexical) decoding than due to the emergence of lexical processing (i.e., whole-word recognition; Wimmer, 1993; Rau et al., 2014; Gagl et al., 2015; see Ziegler and Goswami, 2005, for a theoretical account). Thus, it will be of interest how the children’s individual performance in the pseudoword reading task relates to their capability of parafoveal preprocessing.

Furthermore, we were interested in the relationship between rapid naming (RN) and the preview benefit. In RN tasks, participants are instructed to quickly and accurately name “simple” stimuli, such as objects, digits, or letters. The items are usually arranged in several lines over a page (and thus allowing for parafoveal preprocessing). A wealth of studies reported a correlation between RN and reading performance (e.g., Wolf, 1991; Wolf et al., 2000; Norton and Wolf, 2012). Expectedly, RN is considerably slower in younger readers than in older and more experienced readers. One probable cause for this speed difference in RN could be that the more experienced readers benefit from parafoveal information, whereas the younger readers do so to a much reduced extent. As yet, a direct demonstration of such a relationship is not available. Pertinent evidence, however, was provided by a recent eye movement study which demonstrated that normally developing (Chinese) readers extract information from the parafoveal items in RN, whereas in impaired (i.e., dyslexic) readers parafoveal preprocessing was markedly limited (Pan et al., 2013; see also Jones et al., 2008). A possible explanation for a relationship between parafoveal preprocessing during reading and RN (of digits) is that the increasing automaticity in processing of these (highly overlearned) symbols frees attentional resources which, in turn, can be devoted to the preprocessing of the next (i.e., parafoveal) item. Finally, an additional task assessed visual attention without the requirement of verbal processing. To be specific, we used a child-friendly adaptation of the d2-task (Brickenkamp et al., 2010) which assesses general processing speed, the efficiency of allocating visual attention and visual discrimination.

To sum up, the present eye movement study investigated parafoveal preprocessing during oral sentence reading in children of Grade 2 (with about 1 year of reading experience), Grade 4 (∼3 years) and Grade 6 (∼5 years). We obtained the estimates of the extent of parafoveal preprocessing by means of the novel incremental boundary paradigm (Marx et al., 2015). Our main objective was to assess the developmental course of the preview benefit. In particular, we were interested whether 2nd Grade readers already exhibit beneficial effects of parafoveal preprocessing. Additionally, we assessed how the children’s reading fluency, their efficiency of phonological decoding (i.e., pseudoword reading) and their performance in RN relates to the extent of parafoveal preprocessing during reading.

## Materials and Methods

### Participants

A total of 92 children with normal or corrected-to-normal vision participated in the study. Pupils were recruited from five different schools (from the city of Salzburg and the surrounding area). We obtained parental consent and – on the day of testing – children agreed to participate. For participation, the children received a small gift (e.g., a small ball, soap bubbles). The initial sample contained 31, 30, and 31 children from Grade 2, 4, and 6, respectively. In the present study, we were interested in normal reading development. Thus, children with a below-average and above-average reading speed – defined as a reading quotient of less than 70 (*n* = 1) or more than 130 (*n* = 4; see below) – were excluded from any further analysis. One additional child was excluded from the analysis due to massive data loss in the eye tracking task. The final sample consisted of 29 2nd Graders (15 females; 25 right hander; 5 children had migration background and were bilingual; age: 8;5 *y*;*m*, *SD* = 0;5), 27 4th Graders (13 females; 27 right hander; 7 bilinguals with migration background; age: *M* = 10;4, *SD* = 0;6) and 30 6th Graders (17 females; 30 right hander; 12 bilinguals; age: *M* = 12;6, *SD* = 0;6). The children with a monolingual and bilingual background were comparable in their reading performance, as indexed by the reading speed test (see below; group comparison: *t* < 1).

The experiment was conducted in accordance with the Code of Ethics of the World Medical Association (Declaration of Helsinki) and it was approved by the local ethics committee of the University of Salzburg (“Ethikkommission der Universität Salzburg”).

### Material

#### Reading Fluency

**FIGURE 1:**
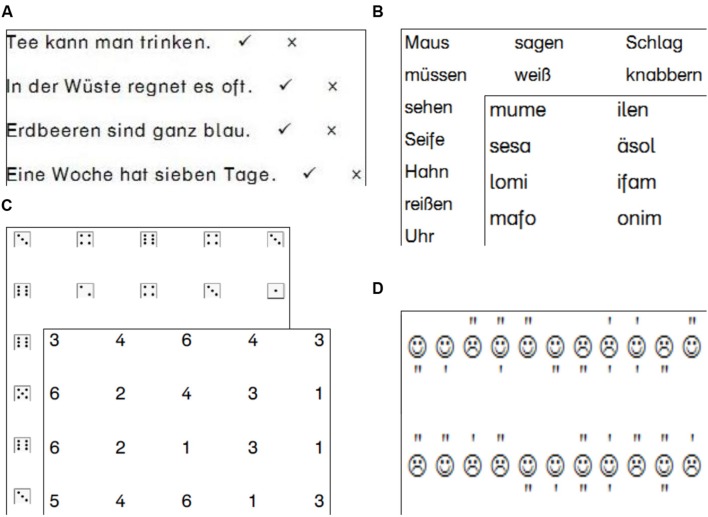
**Illustrations of **(A)** reading speed assessment (silent reading), **(B)** the word and pseudoword reading task (reading aloud), **(C)** the two versions of the rapid naming task, and **(D)** the visual attention task**.

All children conducted a paper–pencil reading speed test. We used the *Salzburger Lese-Screening SLS* [Salzburg Reading-Screening] (Grade 2 and 4: SLS 1–4; Landerl et al., 1997; Grade 6: SLS 5–8; Auer et al., 2005; see **Figure 1A** for an illustration). These tests presented (age-adequate) lists of sentences which either conveys facts of basic knowledge (e.g., “*A week has 7 days*”) or violations of basic knowledge (e.g., “*Strawberries are blue*”). The task of the children was to read the sentences silently and to mark each sentence as correct or incorrect within a time-limit of 3 min. As evident from the examples shown above, the decision as to the “correctness” of the sentences was easy and hence the number of correctly marked sentences is a measure of reading speed. Task performance can be expressed as a reading quotient (*M* = 100, *SD* = 15) based on age-norms from large-sized norming samples. In addition, we conducted a subtest of the *Salzburger Lese- und Rechtschreib-Test* [Salzburg Reading and Spelling Test] (SLRT II; Moll and Landerl, 2010; see **Figure 1B** for an illustration). The subtest required reading aloud words and pseudowords. The measure was the number of correctly read words and pseudowords within a time limit of 1 min.

#### Rapid Naming

For assessing RN ability, we conducted two variants of the RN task. One presented numerals from 1 to 6; the other presented the respective dice faces (see **Figure 1C**). Each RN task consisted of 50 items in a 5-column by 10-row matrix. All RN stimuli were listed in random order with the constraint that adjacent items were not the same. Numerals were presented in an Austrian schoolbook font (20 point). Dices were presented in the same size. The children were familiarized with the test with a short practice array (two rows by five columns per stimulus type). They were timed with a stopwatch while naming the items aloud. The time was then converted to an items-per-minute measure.

#### Visual Attention

The visual attention task we used (i.e., the “Smiley task”) was modeled on the *d2-R* test (Brickenkamp et al., 2010). In the original version, participants are required to mark “d”s which were adorned with two quotation marks, but have to discard similar letters (e.g., “p”s) with two quotation marks or “d”s with only one quotation mark. In our more child-friendly version, the letters were replaced by line-drawings of happy and unhappy faces (i.e., “smileys” and “frownies”; see **Figure 1D**). Children had to mark the smiley faces adorned with two quotation marks. Distractors were smileys and frownies with less or more quotation marks (frownies with 2 marks also served as distractor items). Items were presented in nine lines with 47 smiley faces in each line (30 smiley faces; with an average of 20 right choices) per line. For each line, the children had 20 s whereupon they had to stop and start with the next line. We considered the mean number of correctly marked smiley faces within 1 min as our measure of attention (more specifically, the test assesses general processing speed, serial allocation of visual attention and visual discrimination).

#### Eye Tracking Task

For the eye tracking task we presented 90 sentences in which we embedded one target word per sentence (i.e., *N* = 30 sentences for Grade 2 children; *N* = 60 sentences for Grade 4 children; *N* = 90 sentences for Grade 6 children). The target words were exclusively nouns and had a mean length of five letters (range: 4–6 letters) and a mean frequency (occurrences per million) of 105 according to the SUBTLEX-DE norms (Brysbaert et al., 2011). Note that we used the same sentences as in a previous study from our lab (Marx et al., 2015). The target words were – according to a Latin square design – rotated between the three salience conditions for each Grade. Sentences were constructed in such a way that at least three words preceded and at least one word followed the target word (*M* = 5.4 and 2.5, respectively). The pretarget words were of medium-length and (on average) high-frequency adjectives. Specifically, the mean length of the pretarget word was 5.26 letters (*SD* = 0.84; range: 4–8) and the mean frequency of their lemma-form (i.e., the uninflected form of the word) was 204 per million (word-form: *M* = 85 per million). The length of the experimental sentences ranged from 6 to 12 words (*M* = 8.84, *SD* = 1.11). The sentences were typed in a bold and mono-spaced font. Each character had a width of 8 pixels on the display screen (whose specifications are provided in the “Apparatus” section). From a viewing distance of 50 cm a single character had a width of ∼0.4° of visual angle.

**FIGURE 2:**
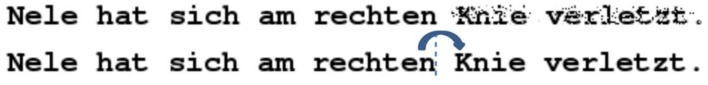
**Illustration of our salience manipulation of the parafoveal preview of the target words.** The upper panel shows a sentence with the medium salience level of the preview. The lower panel illustrates the location of the invisible boundary (dashed line) and the undegraded target and post-target words which appeared after crossing the boundary.

The salience manipulation (i.e., visual degradation) of the stimuli was administered by using the *pixmap-*package (Bivand et al., 2008) and an in-house *R-script*. We had three preview conditions (i.e., parafoveal salience manipulations). In each preview condition all letters of the target and all words thereafter were degraded, that is, a certain amount of black pixels was displaced. The amount of displaced pixels were 0, 10, and 20% for our three levels of degradation (henceforth, we refer to the levels as high, medium, and low salience). An example sentence of our experimental set-up is shown in **Figure 2.**

### Procedure

#### Psychometric Assessment

At first, we administered the reading speed test in the children’s classrooms. The further psychometric assessments (as well as the eye tracking experiment) were conducted over 2 days during which children were seen individually in a quiet area detached from the classroom. On a rotating basis, first and second day procedure and order of tasks were counterbalanced across participants, whereby the psychometric measures lasted approximately 40 min and the eye tracking task lasted approximately 20 min.

#### Eye Tracking

First, we performed a horizontal 3-point calibration routine to familiarize the children with calibrating the eye tracking system. This routine was repeated until the child achieved an average tracking error below 0.5° of visual angle. Then, five familiarization trials for the sentence reading task were administered after which the calibration was repeated – now with a more stringent criterion (average tracking error < 0.3°). Then, we presented the 30, 60, or 90 experimental sentences (dependent on Grade; see section “Eye Tracking Task”). A trial started with a *fixation check*, that is, the presentation of a fixation cross at the left side of the screen (vertically centered). Calibration was repeated when the fixation check failed (but not later than the presentation of 20, 35, or 50 sentences for Grade 2, 4, and 6, respectively). When the system detected a fixation on the fixation cross, the sentence was presented. Display changes were realized with the invisible boundary technique (Rayner, 1975). The boundary was placed at the very end of the pretarget word. Crossing the boundary triggered the presentation of the identical target (and post-target) word(s) – in cases where a high salience preview was presented – or the unmutilated target (and post-target) word(s) – in cases where a medium or low salience preview was presented. The children read the sentences aloud. The experimenter noted reading errors (mostly minor misarticulations, such as, e.g., improper lengthening or shortening of vowels with frequent immediate self-correction by the children).

### Apparatus

Eye movements were recorded monocular for the right eye with a sampling rate of 500 Hz with an EyeLink 1000 (SR Research, Canada). We used the Desktop mount configuration with the “remote” setup which compensates for head movements (by tracking a target sticker on the children’s forehead). The children sat at a viewing distance of approximately 50 cm to the 17 inch CRT-monitor (640 × 480 pixel resolution with a 200 Hz frame rate).

### Eye Movement Measures

We reasoned that the effect of parafoveal preprocessing will be most evident in the initial fixation on the target words. Thus, we considered first fixation (FF) duration as our primary dependent variable. Additionally, we report single fixation duration (SF; i.e, when target words were processed with a SF) and gaze duration (i.e., the sum of all fixations on a target word during first pass reading). Furthermore, we report the initial landing position (ILP), that is, the location of the FF on the target words.

### Data Treatment and Analysis

In total, we administered 5,190 trials (i.e., 29, 27, and 30 children from Grade 2, 4, and 6 read 30, 60, and 90 sentences, respectively; see above). After removal of trials with data loss and outlying fixation times on the target words, 3,860 and 3,851 trials remained for the analysis of FF and gaze duration, respectively. The criteria for outliers were fixations times shorter than 80 ms and longer than 2.65 standard deviation above the individual mean of the participant. For the analysis of SF, we only obtained a total of 1,938 trials, because children seldom processed a word with a SF (see “Results” section). Eye movement data were analyzed by means of linear mixed effects (LMM) modeling using the *lmer*-function of the *lme4*-package (Bates et al., 2015) running within the R environment for statistical computing (R Core Team, 2015). For our global eye movement measures we considered each word except the sentence-initial word and the target word (whose parafoveal preview was manipulated). The model assessed – as fixed effect – the linear effect of Grade and accounted for the random effects of subjects (i.e, the individual children) and items (i.e, the target words). The syntax for this model was *measure* ∼*grade +* (*1| subject*) + (*1| item*). For the analyses of the experimental effect of our salience manipulation on FF, SF, and gaze duration we used a more sophisticated model specification whose syntax was as follows: *measure ∼ salience + grade + salience:grade + (1 + salience + grade + salience:grade—subject) + (1—item)*. The model examined – as fixed effect – the linear effects of Grade and salience and the two-way-interactions between these effects. Besides these fixed effects, the model accounted for the random effects of subjects on the intercept of the model and on the slopes of the salience and Grade effects as well as for random effects of the items. Following standard convention, fixed effects were considered as significant when the corresponding *t*-value was greater than 1.96 (which corresponds to an alpha-level of *p* < 0.05). We *log*-transformed FF, SF, and gaze duration (by the natural logarithm) before entering the analyses, because their distributions were right skewed (the figures, however, presents untransformed data).

## Results

### Reading Rate and Psychometric Measures

Mean task performances as a function of Grade are presented in **Table 1.** The first line of the **Table 1** presents the mean reading quotient of the children from Grade 2, 4, and 6: The groups of children exhibited, on average, normal reading rates (compared to the respective age-norms of *M* = 100 and *SD* = 15). Accordingly, a univariate ANOVA revealed no group differences; *F* < 1.1. In absolute terms, reading rate almost doubled from Grade 2 to Grade 6 as evident from the word-per-minute measure of reading aloud lists of unrelated words; *F*(2,85) = 42, *p* < 0.001. The gain in reading speed was significant between each Grade (*post hoc* pairwise comparisons: *t*s > 4.15, *p*s < 0.001). Likewise, reading aloud lists of unrelated pseudowords showed an improvement with Grade; *F*(2,85) = 25, *p* < 0.001 (*post hoc* pairwise comparisons: *t*s > 2.96, *p*s < 0.01). Furthermore, the number of words-read-per-minute (assessed in our eye tracking experiment; lower section of **Table 1**) increased with Grade; *F*(2,85) = 40, *p* < 0.001. Pairwise comparisons revealed that the difference was significant between each Grade (*t*s > 3.98, *p*s < 0.001). Likewise, the reading accuracy (assessed in our eye tracking experiment) improved with Grade; Kruskall–Wallis *x*^2^ = 17.48, *p* < 0.001. The differences were significant between each Grade (Mann–Whitney *U*s < 265, *p* < 0.04). Furthermore, children became faster in both versions of the RN task; main effect of Grade: *F*(2,83) = 31.90, *p* < 0.001. Improvements – for both versions of the task – were evident between all Grades; *t*s > 2.55, *p*s < 0.02. With regard to differences between the RN versions, the children’s performance was faster for the digit version than for the dice version; main effect of RN version: *F*(1,83) = 241, *p* < 0.001. This difference was more pronounced in Grade 4 and 6 than in Grade 2; Grade by RN version: *F*(2,83) = 9.54, *p* < 0.001. For our measure of visual attention (i.e., the “Smiley task”), we observed a continuous improvement with Grade; *F*(2,85) = 55, *p* < 0.001 (pairwise comparisons: all *t*s > 4.3, *p*s < 0.001).

**Table 1:** Means and standard deviations of the psychometric measures and global eye movements.

	Grade 2		Grade 4		Grade 6	
			
	*Mean*	*SD*	*Mean*	*SD*	*Mean*	*SD*
**Psychometric measures**						
Silent reading [RQ^a^]	103	11	101	12	98	16
Reading aloud words	50	12	71	22	95	21
Reading aloud p-words^b^	33	7	41	13	56	17
Rapid naming						
Dices	79	19	102	16	115	22
Digits	98	25	134	25	153	32
Visual attention	42	6	56	10	69	12
**Eye movement task**						
Words per minute	74	18	102	26	131	28
Reading errors [%]	23	23	10	8	6	5
*N* of fixations per word	1.9	0.3	1.7	0.4	1.5	0.2
Fixation duration [ms]	451	110	360	82	288	45
Saccade length [letters]	3.7	0.8	4.2	0.7	4.6	0.6
Regressions [%]	18	9	19	9	15	6


*^a^Reading Quotient, ^b^pseudowords.*

### Global Eye Movement Measures

As evident from the lower section of **Table 1**, the mean number of fixations per word decreased with Grade; *b* = –0.207, *SE* = 0.040, *t* = –5.13. This reduction was significant between both, Grade 2 and 4; *b* = –0.240, *SE* = 0.093, *t* = –2.58, and Grade 4 and 6; *b* = –0.175, *SE* = 0.079, *t* = –2.20. The mean fixation duration decreased with Grade; *b* = –0.184, *SE* = 0.024, *t* = –7.76, and the difference was significant between Grade 2 and 4, and Grade 4 and 6; *b* = –0.189, *SE* = 0.053, *t* = –3.54 and *b* = –0.180, *SE* = 0.048, *t* = –3.77, respectively. The mean forward saccade length increased with Grade; *b* = 0.462, *SE* = 0.087, *t* = 5.30 (*b* = 0.468, *SE* = 0.192, *t* = 2.43 and *b* = 0.458, *SE* = 0.163, *t* = 2.81 for the Grade 2 – 4 and 4 – 6 comparisons). Finally, there was a linear, but insignificant trend toward fewer regressions with Grade; *b* = –0.019, *SE* = 0.010, *t* = –1.82.

### Target Words

**FIGURE 3:**
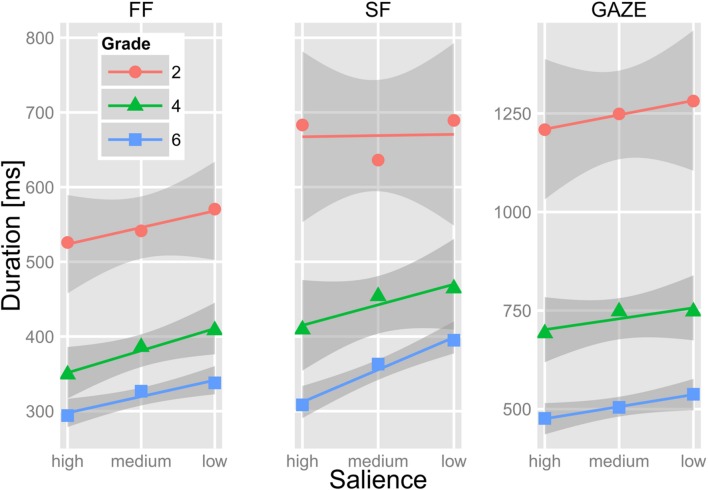
**Mean first fixation (FF), single fixation (SF), and gaze duration on the target word of the children from Grades 2, 4, and 6 in relation to the salience of its parafoveal preview.** The lines show the linear trends of fixation durations in relation to salience. The gray shadings depict 1 SEM as estimated with the *smooth-*function (method = “l m”) of the *ggplot*-package (Wickham and Chang, 2015).

The target words were rarely skipped (*M* < 3.6% for each Grade) and seldom processed with a SF, i.e., in only 12, 20, and 28% of the trials for Grade 2, 4, and 6, respectively. **Figure 3** presents fixation time measures on the target words in relation to our salience manipulation of the target words’ parafoveal preview and Grade. As evident from **Figure 3**, fixation durations became progressively shorter with Grade. This development toward shorter fixation durations was reflected by a main effect of Grade (see **Table 2** for model estimates and the corresponding *t*-values). Critically, all Grades exhibited shorter FF durations for high-salience than for low-salience previews of the target words. For the undegraded (high-salience) previews, the means of FF were 526 ms (*SD* = 112 ms), 349 ms (*SD* = 79 ms), and 294 ms (*SD* = 37 ms) for Grade 2, 4, and 6, respectively. For the low-salience previews, the means were 571 (*SD* = 134), 408 (*SD* = 54), and 338 ms (*SD* = 39) resulting in mean differences of 45, 59, and 44 ms for Grades 2, 4, and 6, respectively. Accordingly, the LMM revealed a main effect of salience but the interaction between salience and Grade was not significant (see **Table 2**).

**Table 2:** LMM estimates of fixed effects (upper part) and estimates of variance (lower part) for first fixation and single fixation duration and gaze duration.

	First fixation duration			Single fixation duration			Gaze duration		
			
Fixed effects	*b*	*SE*	*|t|*	*b*	*SE*	*|t|*	*b*	*SE*	*|t|*
Intercept	6.045	0.046	132.54	6.277	0.065	96.68	6.792	0.070	97.26
Salience	0.063	0.023	2.67	0.056	0.032	1.74	0.037	0.024	1.53
Grade	–0.227	0.031	7.41	–0.303	0.040	7.64	–0.404	0.044	9.23
Salience × grade	0.012	0.014	0.81	0.039	0.018	2.21	0.030	0.015	2.01

**Random effects**	**Variance**	***SD***		**Variance**	***SD***		**Variance**	***SD***	

Intercept: Item	0.007	0.08		0.014	0.12		0.023	0.15	
Intercept: Subject	0.054	0.23		0.121	0.35		0.146	0.38	
Salience	0.015	0.12		0.030	0.17		0.008	0.09	
Grade	0.002	0.04		0.005	0.07		0.005	0.07	
Salience × grade	0.004	0.06		0.006	0.08		0.002	0.05	
Residual	0.145	0.38		0.073	0.27		0.223	0.47	


*Model: log(duration) ∼ salience + grade + salience:grade + (1 + salience + grade + salience:grade | subject) + (1 | item).*

Remember that the children seldom processed the words with a SF and, thus, the analysis of SF duration should not be overrated. In short, **Figure 3** shows that Grade 4 and Grade 6 exhibited shorter SF durations for high-salience than for low-salience previews of the target words. The children from Grade 2 did not exhibit such an effect. Accordingly, the LMM revealed an interaction between salience and Grade; the main effect of salience did not reach significance. Separate LMMs for each Grade revealed significant effect of salience in each Grade. The fixed effects of salience, however, were much higher for the children of Grade 4 (*b* = 0.111, *SE* = 0.027, *t* = 4.18) and Grade 6 (*b* = 0.134, *SE* = 0.108, *t* = 12.42) than for the children of Grade 2 (*b* = 0.073, *SE* = 0.033, *t* = 2.20). It is noteworthy that – as evident from **Figure 3** – SF were, on average, longer than FF (see “Discussion”). Pairwise comparisons (independent of the level of salience) revealed that this difference was significant for each Grade; all *t*s > 5.1 (*df* = 19, 26, and 29 for Grade 2, 4, and 6, respectively), all *p*s < 0.001.

The LMM for gaze duration did not reveal a significant main effect of salience, but a significant interaction between salience and Grade. Separate models revealed that the fixed effect of salience was significant in Grade 4 (*b* = 0.065, *SE* = 0.021, *t* = 3.10) and Grade 6 (*b* = 0.098, *SE* = 0.015, *t* = 6.55). For Grade 2, the effect of salience did not reach significance (*b* = 0.052, *SE* = 0.028, *t* = 1.85).

**FIGURE 4:**
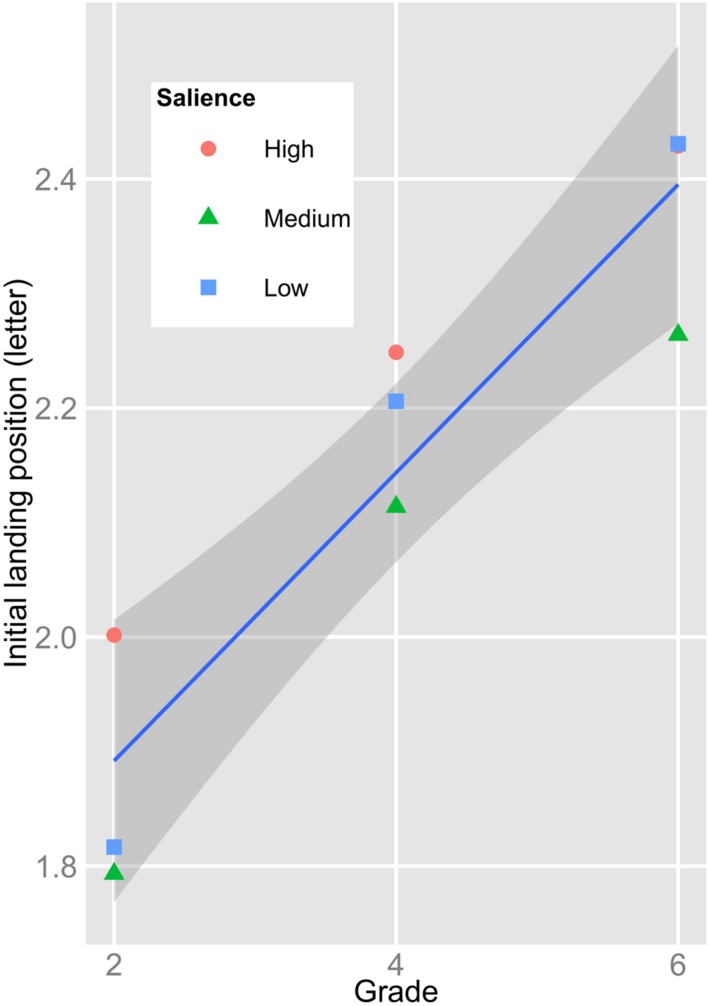
**Mean initial landing position (ILP) on the target words in relation to Grade and the salience of the parafoveal preview of the target words**.

**Figure 4** presents the ILP of the children in relation to Grade and the salience of the parafoveal preview. As evident from **Figure 4**, increasing Grade-level is associated with progressively more rightward fixation locations (i.e., toward the word center); *b* = 0.209, *SE* = 0.079, *t* = 2.63. In absolute terms, however, the increase of ILP was rather small (less than half a letter from Grade 2 to Grade 6). Critically, there was neither a main effect of salience nor an interaction of salience with Grade; both |*t*s*|* < 1.06.

### Correlations of the Psychometric Measures and Parafoveal Preprocessing

**FIGURE 5:**
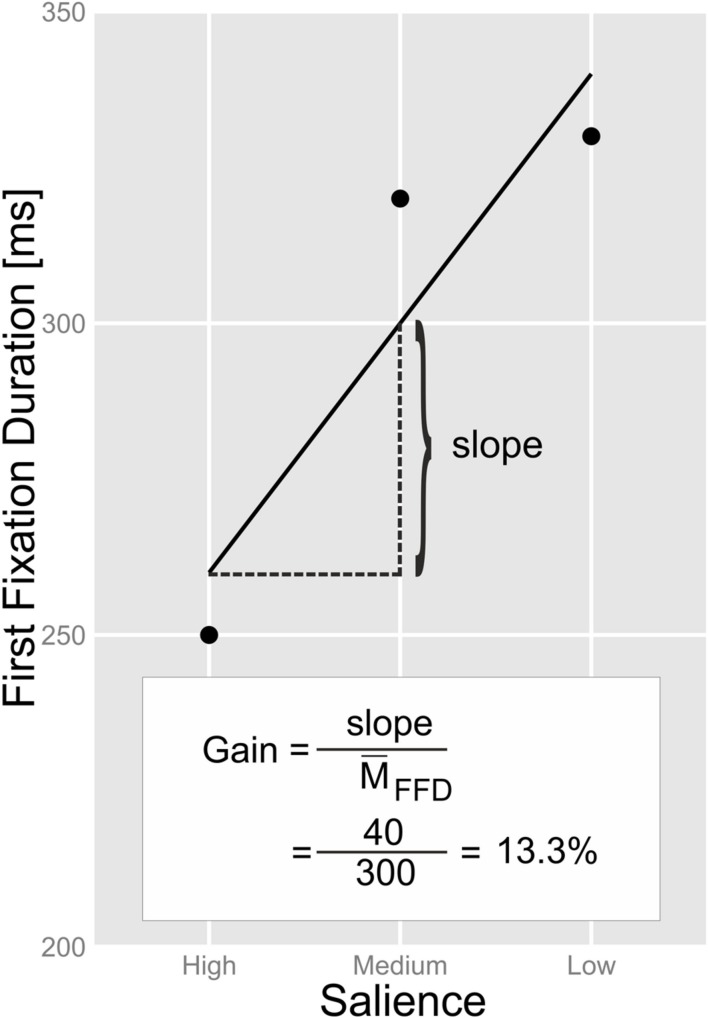
**Exemplary illustration of our procedure of estimating the “gain” that parafoveal preprocessing provides for the subsequent foveal processing of the target words.** The proportion [p] of gain was multiplied by 100 in order to achieve a gain estimate in the unit of percentage.

Our procedure of assessing the association of parafoveal preprocessing with individual differences in the psychometric measures was as follows: we obtained the individual preview benefit of the participants from the random effect of the LMM of FF (by means of the *ranef*-function). The random effect expresses to which degree the slope of the individual participants deviates from the average slope of the whole sample. We then computed the proportional reduction of FF in relation to the salience of the parafoveal preview by dividing the individual slopes of the participants by their mean FF duration (see **Figure 5**).

**FIGURE 6:**
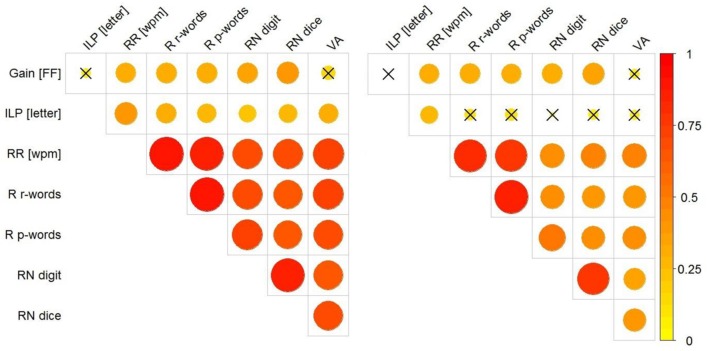
**Correlations (Pearson’s *r*) between the proportional reduction of first fixation duration in relation to the salience of the parafoveal preview (Gain [FF]), the ILP on the target words (ILP), the word-per-minute (*wpm*) rate of reading aloud sentences (RR; obtained from the eye tracking task), the *wpm*-rate of reading aloud lists of words (R r-words) and pseudowords (R p-words), the items-per-minute measure of the two versions of the rapid naming task (i.e., RN digits and dice faces) and the performance in the visual attention task (VA).** The **(Left)** panel presents the correlations irrespective of the Grade-level of the children; the **(Right)** panel shows the correlations after partialling-out Grade. The size of the correlations is represented by the size (and the color) of the circles; correlations of *r* > 0.23 were significant (*p* < 0.05); insignificant correlations are marked with an X. For creating this Figure, we used the *corrplot*-package (Wei, 2013).

**FIGURE 7:**
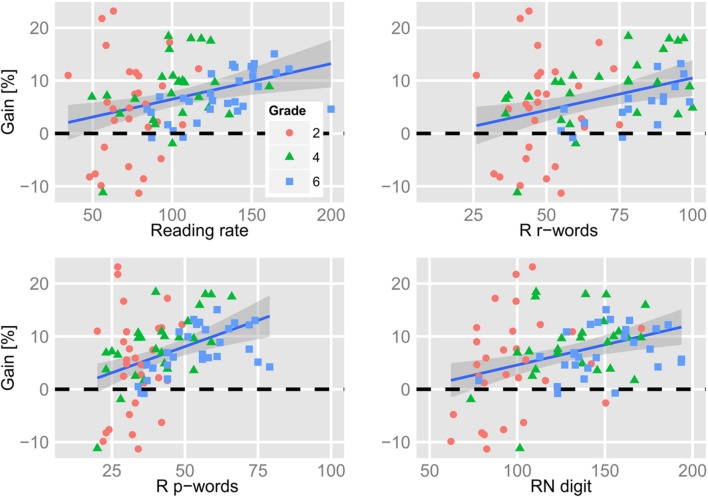
**Relation between the percentage of gain (preview benefit) with psychometric measures (reading rate from the eye tracking task, reading real words, reading pseudowords, and rapid naming digits).** Positive values (i.e., values above the bold dashed line) indicate gain that is the proportional reduction of the first fixation duration in relation to the salience of the parafoveal preview of the target words (see **Figure 5** and main text for details).

**Figure 6** shows the correlation between the following measures: (i) the individual gain that parafoveal preprocessing provided for foveal processing of the target words (i.e., our estimate of the magnitude of the preview benefit; see **Figure 5**), (ii) the ILP on the target words, (iii) the reading rate as expressed by words-per-minute (from the eye tracking experiment), the rate of reading aloud columns of (iv) words and (v) pseudowords, the performance in (vi) the dice-version and (vii) the digit-version of the RN task, and (viii) the performance in the visual attention task (VA; i.e., the “Smiley” variant of the d2-test). The left panel of **Figure 6** shows the correlations for all participants irrespective of Grade; the right panel shows the correlations when we partialled-out the effect of Grade. Contrasting full versus partial correlations gives us indications as to whether an association of a variable with our index of parafoveal preprocessing (i.e., “gain”) reflects “merely” a Grade-related improvement in both measures or whether there is a specific (Grade-independent) relationship. As evident from the left panel of **Figure 7**, the reading rate measures and the performance in the two versions of the RN task were highly (inter-)correlated. Furthermore, reading rates and RN were highly correlated with the performance in the VA task. Partialling-out Grade reduced the size of the correlations of RN and VA with the reading rate measures. Critically, our estimate of the usage of parafoveal information for subsequent foveal word recognition (gain) correlated (moderately) with our various reading rate measures and with RN (see also **Figure 7**). These correlations were significant even when we partialled-out the effect of Grade. The gain due to parafoveal preprocessing was not correlated with the ILP on the target words and not with VA. ILP was reliably associated with reading rate during reading the experimental sentences (i.e., from the eye movement assessment). This association remained significant when we partialled-out Grade. The correlations between ILP and the reading rates for words and pseudowords, RN and VA were insignificant after controlling for Grade.

**Figure 7** shows the relationship between the estimated gain due to parafoveal preprocessing and selected psychometric measures (with the individual scores of the participating children). From the top-right corner to the bottom-left corner, **Figure 7** shows how the gain measure relates to the reading rate from the eye tracking/sentence reading task, the word-list reading task, the pseudoword-list reading task and the RN-digit task. Average reading rates were task-dependent (RN digits > reading words in sentences > reading list of words > reading pseudowords; see **Table 1**). **Figure 7** shows that we observed stable gains due to parafoveal preprocessing when the children read more than 100 words-per-minute in the sentence reading task. For reading lists of words and pseudowords, the respective figures were ∼75 and ∼50 items-per-minute. For RN of digits, we observed relatively stable gains when the children’s rate was greater than 150 items-per-minute. Finally, we assessed which of the four rate measures is the most potent predictor of the ability of gaining parafoveal information from the upcoming word. To this end, we fitted a linear model with the four predictors and submitted this model to the *stepAIC*-function of the MASS-package (Venables and Ripley, 2002). This function performs a stepwise model selection on the basis of the Akaike information criterion (AIC). This analysis revealed that the performance in the pseudoword list reading task is the best predictor of the preview benefit in our sample of German-reading children.

## Discussion

The main objective of the present developmental eye tracking study was to examine when children begin to effectively utilize parafoveal information during reading. In an earlier study from our lab (Marx et al., 2015), we found that children with about 3 years of reading experience (i.e., children in Grade 4 of primary school) exhibited a substantial preview benefit – similar to children with about 5 years of reading experience (Grade 6). Thus, we assumed that parafoveal preprocessing emerges early during reading acquisition. In the present study, we tested children from Grades 2, 4, and 6.

For the assessment of the magnitude of parafoveal preprocessing we used a recently developed paradigm which combines the classical invisible boundary paradigm (Rayner, 1975) from the field of eye movement research with the rationale of the *incremental priming* technique (Jacobs et al., 1995) from the field of visual word recognition. The rationale behind administering this novel technique was that recent evidence indicated that the application of parafoveal masks – which is the traditional approach for estimating the preview benefit in the context of the invisible boundary paradigm – may lead to an overestimation of the preview benefit (Hutzler et al., 2013; Kliegl et al., 2013; Marx et al., 2015). The *incremental boundary* technique (which systematically manipulates the salience of the parafoveal preview of the target words; see “Introduction”) is much less susceptible to such a bias (Marx et al., 2015).

The main finding of the present study is that children from Grades 2, 4 and 6 exhibited substantially shorter FF durations with increasing salience of the parafoveal preview, that is, they exhibited a preview benefit. For FF duration on the target words, the incremental boundary approach (i.e., comparing mean FF duration for high-salience with those of low-salience previews) revealed estimates of the size of the preview benefit of about 45 ms for children of Grades 2 and 6 and for children of Grade 4 the size was about 60 ms. These figures translate to a shortening of fixation duration of about 8% in Grade 2 and of about 15 and 13% in Grade 4 and 6 when preprocessing of a valid (i.e., high salience) preview is possible compared to instances in which parafoveal preprocessing is hindered (by a visually degraded preview). Thus, we found clear evidence of a parafoveal preview benefit on FF duration for all of the Grades.

The instances in which the children processed the words with a SF were rare – even in the most experienced readers of Grade 6 (<30%). The low number of SF cases indicates that our children (learning to read the regular German orthography) achieve visual word recognition primarily due to serial (grapheme–phoneme) decoding – even when they already have considerable reading experience (for similar results and interpretation see Rau et al., 2014 and Gagl et al., 2015). For the children of Grades 4 and 6, however, we observed a preview benefit on SF duration (i.e., reliable effects of our manipulation of the parafoveal preview). Replicating previous findings (e.g., Hawelka et al., 2010), the mean duration of SF were longer than the average duration of FF on the target words. Processing words with a SF has been considered as reflecting whole-word recognition and the prolongation of SF in comparison to FF may reflect the completion of lexical processing, that is, accessing whole-word phonology and word meaning (Hawelka et al., 2010). Thus, parafoveal preprocessing seems to be beneficial for whole-word recognition even if this manner of word recognition is still comparatively rare (as indicated by the small proportion of singly fixated words).

In addition to fixation times, we assessed the ILP on the target words in relation to the Grade-level of the children and to the salience of the preview. The motivation for including this measure was twofold. First, we were interested in the development of the visual scanning behavior during the initial years of reading acquisition. Second, we were interested in the relationship between the extent of parafoveal preprocessing (indexed by the size of the preview benefit) and the saccadic targeting of the upcoming word. With regard to the first aspect, experiments using single word presentation (with French children) revealed that beginning readers quickly acquire an adult-like tendency to fixate at the *optimal viewing position*, that is, (slightly left of) the word center (Aghababian and Nazir, 2000). This shift in targeting the center of a word – as opposed to targeting a word’s initial letters – was previously attributed to the progress from laborious sublexical grapheme–phoneme conversion toward more efficient whole-word recognition (MacKeben et al., 2004; Hawelka et al., 2010; Rau et al., 2015). The efficiency of processing a word by means of sublexical decoding, however, is supposed to be largely dependent on the orthographic depth of the to-be-learned language. To illustrate, a recent eye movement study, which directly compared sentence reading in German (a shallow orthography) and English (a deep orthography; Seymour et al., 2003; Share, 2008) – showed that the German readers relied more on small-unit decoding than their English peers (Rau et al., 2015). Supporting the notion of such a small-unit decoding strategy, recent eye movement studies in regular orthographies reported that beginning readers tend to aim the incoming saccade at the word beginning (Gagl et al., 2015). To illustrate, Gagl et al. (2015) reported – for an experiment with single word presentation – that German-reading children of Grade 2 and 4 fixated the word beginning with little influence of word length on initial fixation location. Likewise, in our previous study (Marx et al., 2015), we found that the ILP of children of Grades 4 and 6 (in a sentence reading task with valid and invalid previews of target words) was at the beginning of the target words. In the present study, we found a significant developmental trend of initial fixation location toward the word center. Furthermore, the ILP was reliably correlated with the reading rate (even when the Grade-level was partialled-out). The size of the Grade effect, however, was – in absolute terms – small (half a letter from Grade 2 to Grade 6). Thus, our finding conforms to the notion that the progress from grapheme–phoneme conversion toward whole-word recognition proceeds slowly in regular orthographies.

With regard to our second interest, we found no association of the ILP with the extent of parafoveal preprocessing. This was even the case, when we correlated these two measures irrespective of Grade (i.e., without partialling-out Grade). The absence of an association between the preview benefit and the ILP conforms to the assumed decoupling of oculomotor control and visual attention (as it is implemented, for example, in the E-Z Reader model of eye movement control during reading; Reichle et al., 1998, 2003). In the conceptualization of the E-Z reader model, the processing of the length of the next word is considered as a basal visual, pre-attentive process. Accordingly, we did not find an association of the ILP with our measure of visual attention (i.e., our variant of the d2-test which assesses the serial allocation of visual attention and visual discrimination) after accounting for Grade-level effects (i.e., after partialling-out age-related improvement in visual attention). The mechanism that oculomotor control and visual attention operates independently can explain the fact that mature readers frequently skip words during reading. The fact that we did not find an association between the amount of parafoveal preprocessing and saccadic aiming in beginning readers could indicate that the functional separation of visual attention and oculomotor planning is already in place during reading acquisition (when word skippings are still very rare).

### The Association of the Parafoveal Preview Benefit with Rapid Naming and Pseudoword Reading

We administered two versions of the RN task, that is, a “standard” version which required the naming of digits (ranging from 1 to 6) and an equivalent version in which we substituted the Arabic numerals with the corresponding dice faces. The rationale for the administration of these two versions was that we assumed that the children of Grade 2 (with only about 1 year of formal education) might not yet exhibit automaticity in processing (in future over-learned) orthographic representations (i.e., the Arabic numerals). The ensuing expectations were that (i) the children from Grade 2 would exhibit a more similar performance in the two RN versions, whereas the older children would perform better in the digit version and (ii) that the association of RN of digits may become stronger with increasing Grade-level. A recent eye movements study by Pan et al. (2013) indeed showed that the eye-voice span is larger during RN of digits than during RN of dice faces (which was interpreted as reflecting the higher automaticity of processing Arabic numerals). Moreover, this effect was markedly more pronounced in typically developing readers than in dyslexic readers (indicating a less automatized processing of Arabic numerals in the latter group). The present findings conform to the notion of heightened automaticity for over-learned orthographic symbols. In each Grade, the children performed better in the digit-version than in the dice-version of the RN task, but the difference was more pronounced in the higher Grades. However, the performance in both versions was associated equally with our estimate of parafoveal preprocessing and this association did not depend on reading experience (i.e., Grade level). The similar association of RN dice faces and digits with parafoveal preprocessing may reflect the shared requirement of coordinating the serial allocation of visual attention (in the direction of reading) and accessing a phonological representation as figured by the visual scanning hypothesis of the relationship of RN with reading (e.g., Kuperman et al., 2016).

The best predictor of parafoveal preprocessing was the children’s performance in the pseudoword reading task. The task assessed the children’s efficiency of phonological decoding. As aforementioned, the developmental transition from sublexical decoding to (lexical) whole-word recognition seems to be a slow process in regular orthographies (e.g., Rau et al., 2014) and hence the improvement in reading rate with increasing experience is – at least partly – due to a gain in the efficiency of phonological decoding (e.g., Wimmer, 1993; Gagl et al., 2015). The present finding adds to this notion by showing that children who excelled on the pseudoword reading task exhibited the largest preview benefit.

### Limitations and Future Directions

One could conceive the present study’s requirement of reading aloud as a limitation for studying the development of the preview benefit, because reading aloud may reduce the extent to which readers engage in parafoveal preprocessing. To illustrate, Ashby et al. (2012) found – in adult participants – that the preview benefit is diminished in oral reading compared to silent reading. However, silent reading is unusual for children – particularly in the early years of primary schools. Another limiting issue, one could argue, is the high variance in the performance of the children from Grade 2. The variance in our dependent measures was much lower in Grade 4 and 6. This pattern conforms to the prediction of, for example, the rate-amount model (Faust et al., 1999) that increasing average efficiency is accompanied with a reduction in variance. To account for a global factor such as general processing speed (e.g., Zoccolotti et al., 2008) was, however, beyond the scope of the present study.

With regard to future directions, the present study (together with the Marx et al., 2015 study) showed that the incremental boundary technique is an adequate tool for studying the emergence and the development of parafoveal preprocessing in developing readers. Future studies may apply the technique to study further aspects of parafoveal preprocessing (for which the evidence is, as yet, based primarily on samples of adult readers). Such aspects are, for example, the relative importance of a word’s initial versus its final letters for parafoveal preprocessing (e.g., Briihl and Inhoff, 1995; Gagl et al., 2014) or the effect of foveal load on the preview benefit (Henderson and Ferreira, 1990).

## Conclusion

The present study provides information as to when parafoveal information is effectively utilized during oral sentence reading. Overall, the findings reveal that children with about 1 year of reading experience start to utilize parafoveal information for subsequent foveal word recognition. However, we observed an association of the preview benefit with reading fluency (indexed by the word-per-minute reading rate) – which substantially overlapped between Grades. Thus, the individual reading competence seems to be the more important constituent of the effective use of parafoveal information for subsequent foveal word recognition than reading experience as indexed by Grade-level. The best predictor of parafoveal preprocessing in our sample of children learning to read a regular orthography was their performance in a pseudoword reading task assessing the efficiency of phonological decoding: The best decoders exhibited the greatest preview benefit.

## Author Contributions

CM performed the experiment, CM and SH analyzed the data. CM, SH, and SS wrote the manuscript. CM and SS prepared the figures, CM, FH, and SH conceived the experiment. All authors reviewed the manuscript.

## Conflict of Interest Statement

The authors declare that the research was conducted in the absence of any commercial or financial relationships that could be construed as a potential conflict of interest.
